# Evaluation of the Bacterial Diversity in the Human Tongue Coating Based on Genus-Specific Primers for 16S rRNA Sequencing

**DOI:** 10.1155/2017/8184160

**Published:** 2017-08-20

**Authors:** Beili Sun, Dongrui Zhou, Jing Tu, Zuhong Lu

**Affiliations:** ^1^State Key Laboratory of Bioelectronics, Southeast University, Nanjing 210096, China; ^2^Key Laboratory of Child Development and Learning Science, Ministry of Education of China, Southeast University, Nanjing 210096, China

## Abstract

The characteristics of tongue coating are very important symbols for disease diagnosis in traditional Chinese medicine (TCM) theory. As a habitat of oral microbiota, bacteria on the tongue dorsum have been proved to be the cause of many oral diseases. The high-throughput next-generation sequencing (NGS) platforms have been widely applied in the analysis of bacterial 16S rRNA gene. We developed a methodology based on genus-specific multiprimer amplification and ligation-based sequencing for microbiota analysis. In order to validate the efficiency of the approach, we thoroughly analyzed six tongue coating samples from lung cancer patients with different TCM types, and more than 600 genera of bacteria were detected by this platform. The results showed that ligation-based parallel sequencing combined with enzyme digestion and multiamplification could expand the effective length of sequencing reads and could be applied in the microbiota analysis.

## 1. Introduction

The complex microbial flora living on or within the human body has long been proposed to contribute to the human health as well as disease [1–8] (Eckburg et al., 2005). Using culturing or unculturing methodology, over 25,000 bacterial phylotypes and over 700 prevalent taxa at the species level have been identified in the oral microbiome, colonizing in the oral cavity including teeth, gingival sulcus, tongue, cheeks, palates, and tonsils [9–20] (Keijser et al., 2008; Nasidze et al., 2009).

As a reservoir for oral microorganisms, food scraps, saliva, and shed epithelial cells, the human tongue has been investigated showing significant association with the microbial communities of the gut and diseases such as gastritis or halitosis [11, 21–24]. In traditional Chinese medicine (TCM), according to the color and thickness, the human tongue coating can be divided into a few types consisting of thin-white coating, thick-white coating, sticky-white coating, thin-yellow coating, and so forth [25]. The color and shape of tongue coating may reflect the composition of the bacteria colonizing the tongue dorsum. Several researches have considered the association between tongue coating microbiome and traditional tongue diagnosis [26–29]. Jiang et al. investigated 19 gastritis patients with a TCM Cold Syndrome or TCM Hot Syndrome tongue coating and 8 healthy controls by Illumina paired-end, double-barcode 16S rRNA V6 tag sequencing. Han et al. sequenced the V2–V4 region of 16S rRNA gene by pyrosequencing to investigate the tongue coating microbiome in patients with colorectal cancer and healthy controls. Their results indicated that the richness of the bacterial communities in the patients with thin tongue coating and healthy controls was higher than in the patients with thick tongue coating.

In the past few years, pyrosequencing of the 16S rRNA gene and sequencing-by-synthesis of metagenomics have been the widely applied technologies in the study of microbial communities. [30–39]. Pyrosequencing technology has the benefits of relatively long length of sequencing read and the drawbacks of high reagent cost and high error rates in homopolymer repeats. Metagenomic analysis requires deep sequencing data mining and large amounts of sequencing reads for gene assembly and annotation [24, 27, 40]. Ligation-based sequencing technology provides inherent error correction by two-base encoding, which makes the platform much more accurate [34]. Due to the short sequencing length, this methodology has a few restrictions in the microbiome diversity research.

In the present study, we develop an effective approach using multigroup amplification and massively parallel ligation-based sequencing technology to determine the bacterial diversity. In addition, we compare the tongue coating microbial diversities of different TCM tongue coating types using this method.

## 2. Materials and Methods

### 2.1. Samples

**Figure 1:**
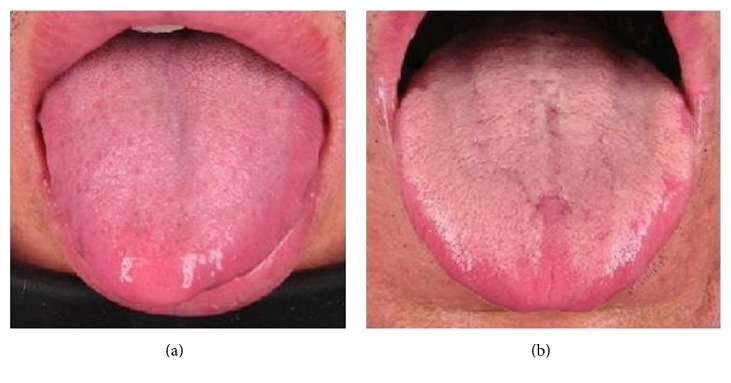
Thin-white tongue coating type (a) and white-greasy tongue coating type (b) in TCM theory.

Tongue coating samples were collected from two groups of lung cancer patients in Nanjing Chest Hospital. Each group represented a specific TCM tongue coating type: the thin-white type and the white-greasy type (Figure 1). A total of 13 subjects were collected in the morning and all the volunteers had no breakfast before sampling. All subjects had no oral inflammation and had refrained from brushing teeth and drinking colored beverage for 16 hours before testing. The tongue coating samples were collected with sterilized cotton swabs, mixed with 1 ml of Ringer's solution, and stored in −20°C immediately until extracting the total microbial genome DNA. All the 13 samples were investigated by DGGE analysis (data not shown). Based on the DGGE results, the bacteria composition was significantly different between different tongue coating types. Six typical samples from two tongue coating types were chosen for thorough parallel sequencing, and the clinical parameters of subjects were shown in Table 1.

### 2.2. Multiple Group PCR Primers Design

**Figure 2:**
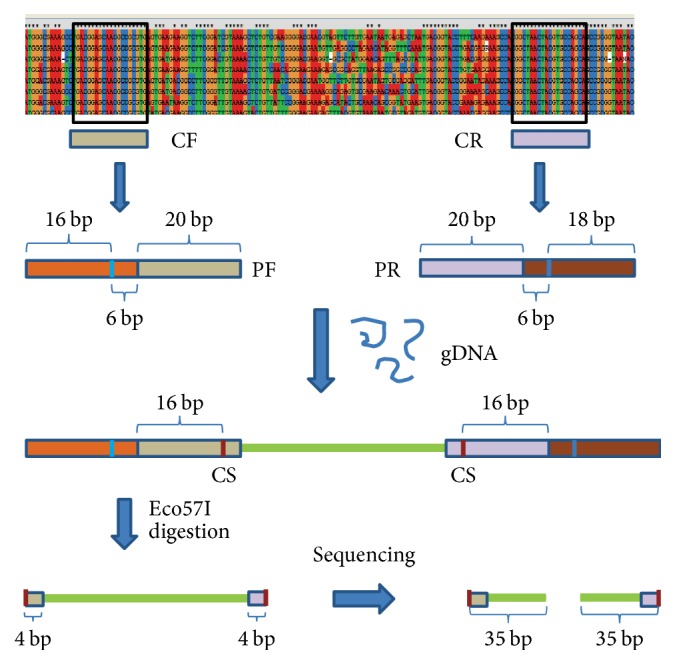
Schematic diagram of the primer design and library preparation. All the 16S rRNA gene sequences from the same genera were aligned to generate 20 bp of the conserved forward sequence (CF) and conserved reverse sequence (CR) flanking the variable region. The forward primer (PF) and reverse primer (PR) were designed by adding 6 bp of Eco57I recognition site and 16/18 bp of auxiliary sequence at the 5′ end of CF and CR. The microbial genome DNA of each subject was amplified by all the pairs of primers from different genus, producing the amplicons containing the Eco57I cutting site (CS) which was 16 bp downstream of the recognition site. Followed by Eco57I digestion, products generated by all the genus-specific primers, conserving 4 bp of each primer sequence, were mixed together to produce the sequencing library. The results of ligation-based sequencing were millions of reads, which were the first 35 bases at the 5′ end from either forward strand or reverse strand of library.

The multigroup PCR primers were designed based on Human Oral Microbiome Database (HOMD) (http://www.homd.org/). The 16S rRNA gene sequences of a genus containing 4 species at least in HOMD were picked out as a group and aligned by MEGA (v 4.0.2). Totally, 421 species belonging to 27 genera were selected. The conserved regions flanking the variable region can be used for the multigroup PCR primers design. A specific group primer consisted of 22 bp of sequences containing Eco57I recognition site and 20 bp of conserved region sequences (Figure 2).  Table 2 indicates all the 26 pairs of group primers generated.

### 2.3. DNA Extraction, Amplification, and Ligation Sequencing Library Preparation

All the samples were centrifuged and resuspended in 1 ml of 10x TE buffer (pH 8). The suspension was mixed with 100 *μ*l of lysozyme (200 mg/ml) and incubated at 37°C for 1 h. Lysis solution containing 50 *μ*l of SDS (10%, v/v) and 20 *μ*l of Proteinase K (20 mg/ml) was added and the mixture was incubated at 55°C for 3 h. The total bacterial genome DNA was obtained by phenol-chloroform extraction and ethanol precipitation.

The PCR amplification was carried out separately by each of group primers for each sample with the same condition except for the annealing temperature. All the PCR reactions were performed in a final volume of 25 *μ*l containing 1x PCR buffer (Takara), 2.5 mM MgCl_2_, 200 *μ*M each dNTP, 2.5 U of Taq polymerase, 20 ng of template DNA, and 1 *μ*M each forward and reverse primer. The PCR mixtures were initially denatured at 95°C for 3 min, followed by 30 cycles: 45 s at 94°C, 45 s at (*T*_m_ − 5)°C, and 45 s at 72°C. The PCR amplicons were digested using the enzyme Eco57I (Fermentas, Burlington, Canada) according to manufacturer's instructions. The expected fragment DNA was acquired by agarose gel electrophoresis and purified by QIAEX II Gel Extraction Kit (Qiagen, Hilden, Germany). PCR amplicon mixture was prepared by pooling approximately equal amounts of recovered fragments from the same sample. After blunting the 3′ protruding termini of mixtures by T4 DNA polymerase, four sequencing libraries were constructed for the ligation-based sequencing system.

### 2.4. Massively Parallel Ligation Sequencing

In order to maximize the sequencing capacity and simplify the workflow of sample preparation, all the samples were operated in a single sequencing run with barcodes added. Six barcodes were ligated to the 3′ end of the library templates with T4 DNA ligase as unique adaptors (Table 1).

SOLiD P1 adaptors (5′-CCACTACGCCTCCGCTTTCCTCTCTATGGGCAGTCGGTGAT-3′) were ligated at the 5′ end of templates, followed by standard SOLiD library preparation protocol.

### 2.5. Construction of Reference Sequences

Due to the characteristic of 2-base encoding in SOLiD sequencing, the sequencing reads are in color-space format “0, 1, 2, 3,” which stands for the permutation of the adjacent bases. The brief data processing pipeline in our study was designed as Figure 2 shows, because the novel microbial analysis methods are inappropriate. A total of 1,049,433 unaligned 16S rDNA sequences of both bacteria and archaea were downloaded from Ribosomal Database Project (RDP) (http://rdp.cme.msu.edu) resource (release 10, update 13). Using in silico analysis of amplification by the designed primers and endonuclease digestion, 26 groups of fragments were selected. Repetitive sequences were moved to construct 26 groups of reference sequences (REF-DB). Taxonomies of REF-DB (TAXA-DB) were assigned using the original full-length 16S rRNA gene sequences by the RDP online classifier (http://rdp.cme.msu.edu/classifier/classifier.jsp) at 80% confidence cut-off. If the reference was yielded from more than one full-length 16S rRNA gene sequence, each of the original sequences was assigned separately. From the genus level, the taxonomy shared by three-fourths majority of the full length sequences was defined as the taxonomy of the reference. Otherwise, taxonomy of higher level was compared until the domain level.

### 2.6. Analysis of Sequencing Data

Sequencing reads were split into six samples according to the 4-mer barcodes in P2 adaptor. In color space, all the samples were aligned separately to REF-DB by Corona Lite Program (v4.2, http://solidsoftwaretools.com) with up to 3 color-space mismatches. For unique reads (uniquely placed matches), a few steps of filtering were performed. Firstly, the matching position should be at the 5′ end of the reference sequence (forward-strand matching) or reverse compliment (reverse-strand matching). Meanwhile, the top four positions of reads were checked with one mismatch in color space to filter out nonspecific amplification or false endonuclease-digested products. Secondly, the abundance of reads matching “forward-strand” and “reverse-strand” references was counted separately and the reads with less than 5 counts in both strands were discarded. Finally, Operational Taxonomic Units (OTUs) were identified according to matched sequences in the REF-DB and corresponding taxonomy information in TAXA-DB. For the nonunique reads that matched the reference in more than one location, all of the matched references were assigned at the phylum level according to TAXA-DB. A matched read was assigned to a specific taxonomy, while all the references it matched belonged to accordant phylum. Otherwise, the read was denoted as “undefined.” Both the unique and nonunique matched reads were summarized by a set of Perl scripts.

### 2.7. Phylogenetic Analysis

The original full-length sequences of matched OTUs were aligned using ClustalW, and a relaxed neighbor-joining tree was built by PHILIP 3.68. Weighted and unweighted UniFrac were run using the resulting tree and environment annotation of number of hits. PCA was performed on the resulting matric of distances between each pair of samples.

### 2.8. Prediction of Metagenome from 16S rRNA Gene Data

The PICRUSt project aims to support prediction of the unobserved character states in a community of organisms from phylogenetic information about the organisms in that community. This program was used to predict metagenome abundance from 16S rRNA gene data.

## 3. Results

### 3.1. Sequencing Performance of SOLiD Reads

One-quarter of slide was used to perform the ligation-based sequencing, and 84,399,432 reads of 35-base length (2.75 gigabases in total) were captured after removing sequences of insufficient quality. Splitted by 4-mer barcodes, a total number of 75,115,494 reads (89%) were generated (Table 3). The difference of sequencing throughput among six samples was probably due to the sample quality or the procedure of library preparation.

### 3.2. Taxa Assignment of the Sequencing Reads

Since SOLiD system employs 2-base encoding and color-space strategy, a single color change is a measurement error, two adjacent color changes may result from a single nucleotide variation in base space, and three color-space mismatches might imply two adjacent variants compared to reference. We used up to three color changes in 35-base length as the alignment parameter, which implied at most two nucleotide mismatches. Consequently, the sequence similarity was more than 94%, which corresponded to the genus level classification. Aligned to REF-DB, matched and uniquely matched reads were summarized in Table 3.

To increase the accuracy of taxonomy assignment, two steps of validation were performed before OTUs definition as Methods described. Altogether, we discarded 33.75% of the uniquely matched reads, leaving 647,375 sequencing reads for taxonomy analysis. Compared to TAXA-DB, number of OTUs was identified, ranging from 409 to 669 for six samples, respectively. Most of the OTUs (more than 93%) could be assigned at the genus or lower level, while a very small amount of OTUs was just assigned at the phylum or higher level (Table 4).

### 3.3. Microbiome Diversity of the Tongue Coating Samples

**Figure 3:**
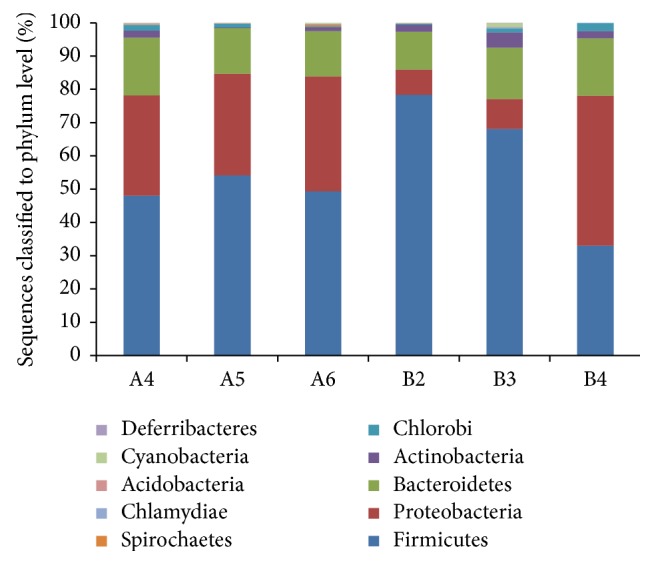
Relative abundances of taxonomy classification of the tongue coating microbiome at the phylum level in the six tongue coating samples.

**Figure 4:**
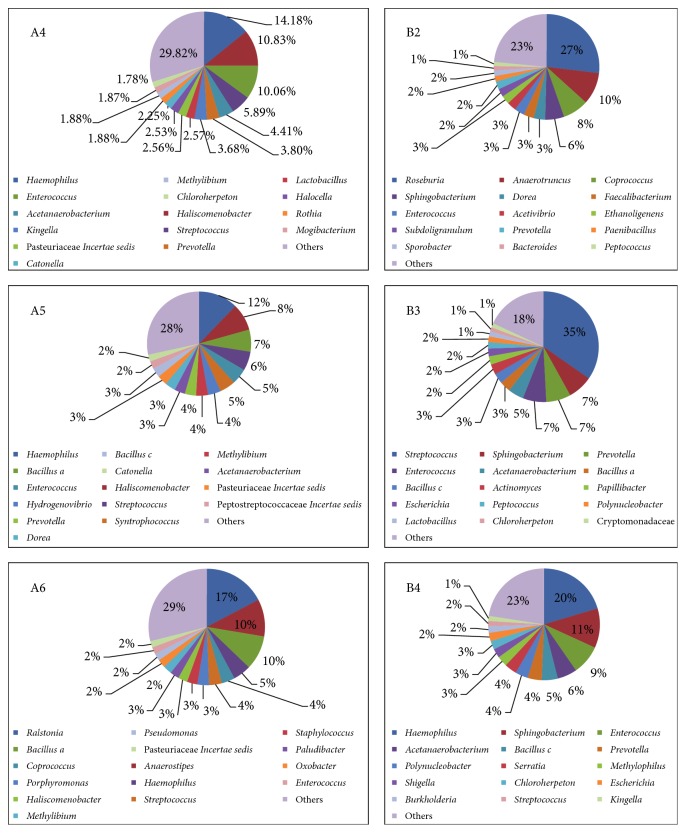
Dominant genera (top 15) assigned in the six samples.

The dominant phyla (relative abundance > 1%) across all samples were Firmicutes (55.19%  ± 16.00%), Proteobacteria (26.11%  ± 14.85%), Bacteroidetes (14.80%  ± 2.36%), Actinobacteria (2.03%  ± 1.45%), and Chlorobi (1.17%  ± 0.79%). Ultimately, we identified 209 genera from the six samples, and the abundance of each genus was defined in Methods. As shown in Figure 3, 92 genera were assigned to phylum Firmicutes, with the maximum abundance as well. Phyla Proteobacteria and Bacteroidetes contained some dominant genera in our results, while only 68 and 30 genera were assigned to these two phyla separately. The fifteen most abundant genera of each sample were illustrated in Figure 4, with total abundance ranging from 70.2% to 82%. There were 163 genera assigned from 79,762 reads for sample A4, and the most abundant genera were* Haemophilus* (13.38%, Proteobacteria),* Haliscomenobacter* (10.23%, Bacteroidetes),* Enterococcus* (9.50%, Firmicutes),* Streptococcus* (5.56%, Firmicutes), and* Acetanaerobacterium* (4.17%, Firmicutes). For sample A5, 134 genera were identified from 41,580 reads, having the most abundant genera of* Haemophilus* (11.40%),* Haliscomenobacter* (7.81%),* Bacillus a.* (6.40%, Firmicutes),* Streptococcus* (5.50%), and* Enterococcus* (4.62%). There were 141 genera assigned from 242,085 reads for sample A6, and the most abundant genera were* Ralstonia* (17.31%, Proteobacteria),* Anaerostipes* (10.32%, Firmicutes),* Bacillus a* (9.53%, Firmicutes),* Haemophilus* (4.96%), and* Coprococcus* (3.69%, Firmicutes). For white-greasy tongue coating type samples, for sample B2, 119 genera were found within 155,093 reads, and the dominant genera were* Roseburia* (26.91%, Firmicutes),* Anaerotruncus* (9.8%, Firmicutes),* Coprococcus* (7.91%),* Sphingobacterium* (5.81%, Bacteroidetes), and* Dorea* (3.48%, Firmicutes). For sample B3, 128 genera were assigned from 47,717 reads, with the largest proportion genera of* Streptococcus* (34.25%),* Sphingobacterium* (7.14%),* Prevotella* (7.08%, Bacteroidetes),* Enterococcus* (6.79%), and* Acetanaerobacterium* (4.50%). For sample B4, 143 genera were found within 81,138 reads, and the dominant genera were* Haemophilus* (19.03%),* Sphingobacterium* (10.65%),* Enterococcus* (7.94%),* Acetanaerobacterium* (5.31%), and* Bacillus c* (4.32%, Firmicutes). Overall, the dominant microbial phyla of the three thin-white tongue coating samples were Firmicutes, Proteobacteria, and Bacteroidetes, while the white-greasy samples B2 and B3 had the second abundant phylum of Bacteroidetes, and the sample B4 had the most abundant phylum of Proteobacteria.

In order to maximize the high-throughput usable data for deep analysis, both the uniquely and nonuniquely matched reads were classified at phylum level. The percent of assigned reads was ranging from 58.8% to 65.6%. Compared with the results based on unique reads, the proportion of phyla Firmicutes and Actinobacteria had dramatically increased (data not shown).

### 3.4. Shared Genera of the Same Tongue Coating Type Samples

We compared the shared genera within the same tongue coating type samples. There were 117 genera shared between the thin-white tongue coating type samples. Of these genera, 58 were from Firmicutes, 37 genera were from Proteobacteria, and 12 genera belonged to Bacteroidetes. For the white-greasy tongue coating type samples, 96 genera were observed to be shared. 48 genera belonged to Firmicutes, 28 genera belonged to Proteobacteria, and 11 genera belonged to Bacteroidetes, respectively.

### 3.5. Comparison of Bacterial Diversity between Different Tongue Coating Type Samples

**Figure 5:**
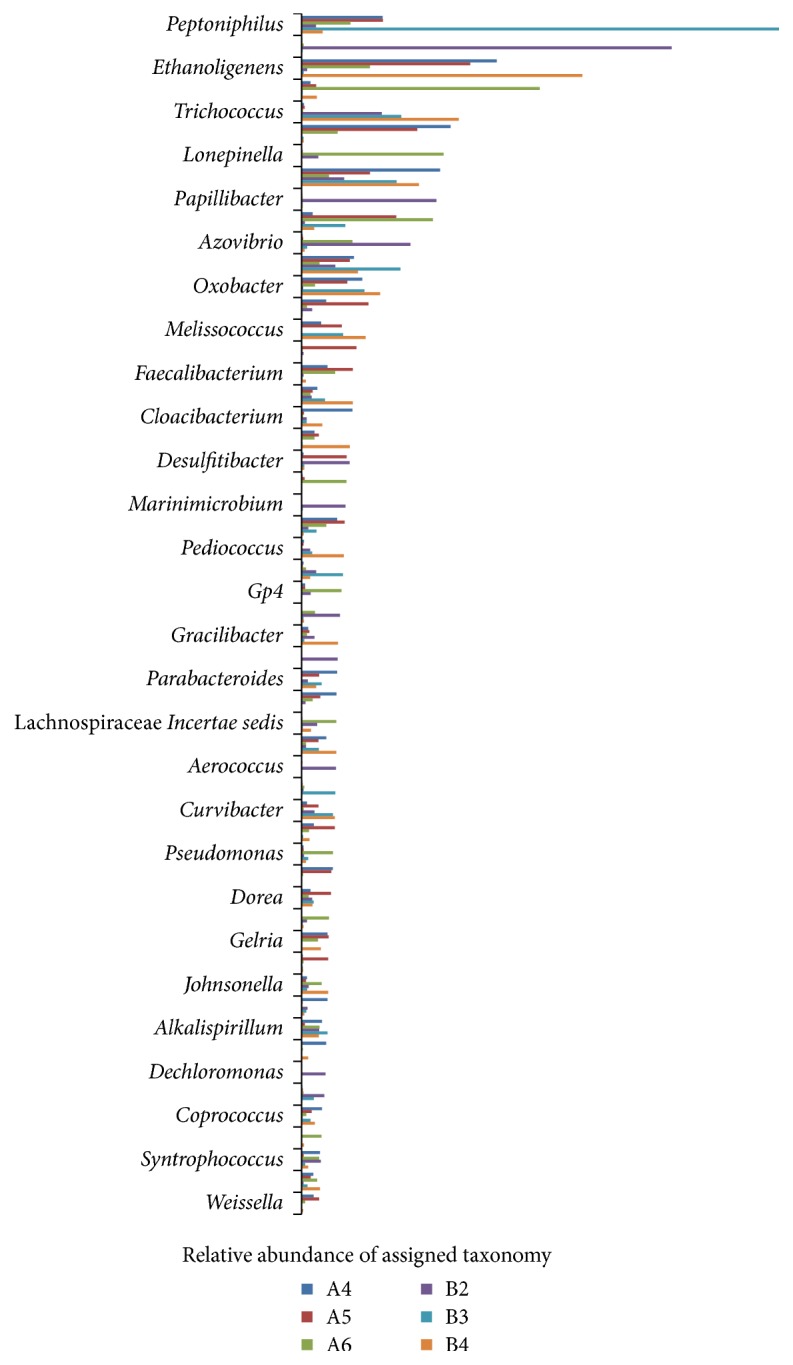
Relative abundance of assigned taxonomy at genus level shared in the six tongue coating samples.

**Figure 6:**
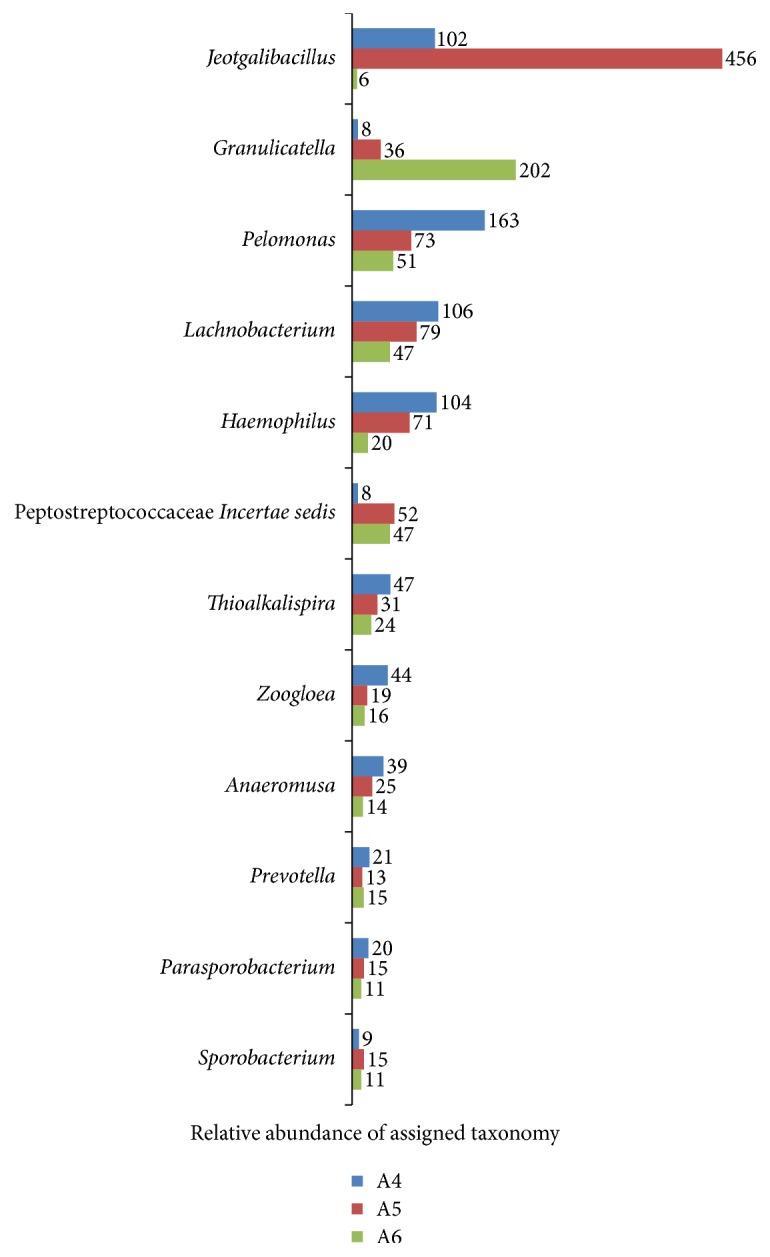
Relative abundance of assigned taxonomy at genus level shared only in the thin-white tongue coating samples.

**Figure 7:**
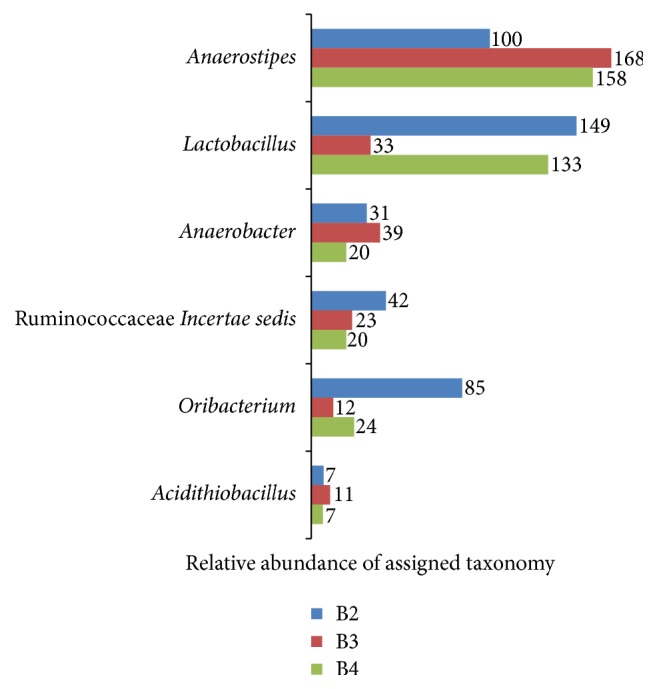
Relative abundance of assigned taxonomy at genus level shared only in the white-greasy tongue coating samples.

There were 77 genera shared across the six samples (Figure 5). 35 genera belonged to Firmicutes, 23 genera belonged to Proteobacteria, and 8 genera belonged to Bacteroidetes. We compared the genera observed in one tongue coating type but not the other.  Figure 6 showed the genera that appeared in all the thin-white tongue coating type but existed in none white-greasy tongue coating type sample. A total of 12 genera were observed only in the thin-white tongue coating type samples:* Pelomonas, Haemophilus, Thioalkalispira*, and* Zoogloea* (Proteobacteria);* Jeotgalibacillus, Granulicatella, Lachnobacterium, Peptostreptococcus, Anaeromusa, Parasporobacterium, *and* Sporobacterium* (Firmicutes); and* Prevotella* (Bacteroidetes).  Figure 7 showed the genera that appeared in all the white-greasy tongue coating type but did not exist in all the thin-white tongue coating type sample. The white-greasy tongue coating type samples were specifically unique for 6 genera:* Anaerostipes*,* Lactobacillus*,* Anaerobacter, *Ruminococcaceae* Incertae sedis*, and* Oribacterium* (Firmicutes) and* Acidithiobacillus* (Proteobacteria). Analyzed by Student's *t*-test, 19 genera performed significantly different between two tongue coating types (Table 5).

**Figure 8:**
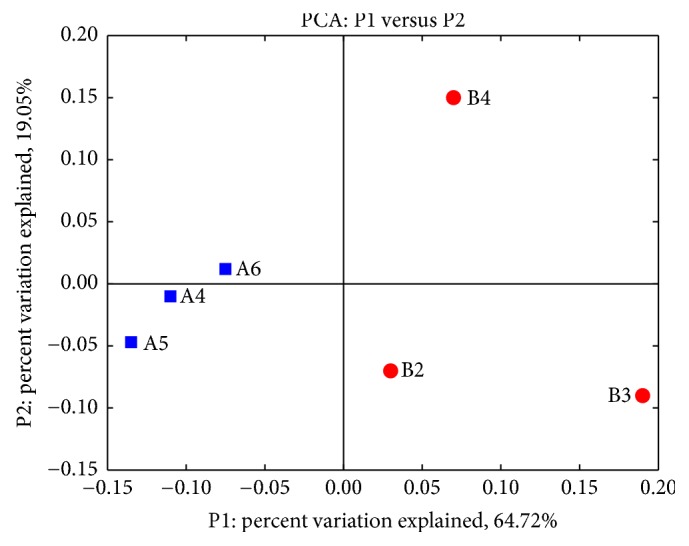
Principal component analysis of microbial diversities of thin-white tongue coating patients (blue squares) and white-greasy tongue coating patients (red circles).

Analyzed by UniFrac software, the thin-white tongue coating type and white-greasy tongue coating type were observably different at the genus level as shown in principal component analysis (PCA) plot (Figure 8). The three thin-white tongue coating subjects had relatively similar microbial diversity (Bonferroni-corrected *P* value is 0.25), while three white-greasy tongue coating subjects behaved clear deviation (Bonferroni-corrected *P* value, all >0.5).

### 3.6. Metagenome Prediction

PICRUSt was used to predict a microbial community metagenome based on 16S gene data. PICRUSt results were then analyzed using LEfSe to identify microbial functions that were significantly different in their relative abundance among groups. The top of differentially abundant bacterial functions were “oxidative phosphorylation,” “ribosome,” “amino sugar and nucleotide sugar metabolism,” and “secretion system.”

## 4. Discussion 

In this study, we explored a method to detect the microbial diversity using 16S rRNA gene by high-throughput SOLiD sequencing system. The ligation-based system features 2-base encoding, which is a proprietary mechanism that interrogates each base twice. The 2-base encoding algorithm filtered raw errors after sequencing, providing built-in error correction. The output of sequencing reads is in the format of color space, which means that the output reads must align to color-space reference. Although the short length is the main drawback of SOLiD platform compared to other sequencing technologies, our method could extend the effective length of sequencing read to more than 100 bp. First, the sequencing length was extended to 51 bp, by adding 16 nucleotides digested by Eco57I. Second, concerning that the two strands of library DNA could be sequenced in 5′ to 3′ direction, the usable sequencing length was equivalent to 102 bp, which was close to the length of one hypervariable region of 16S rRNA gene. Meanwhile, the validation of first four sequenced nucleotides, which were conserved from the designed primers, could enhance the sequencing accuracy in the validation step.

To evaluate the effect of this methodology, six tongue coating samples from different TCM tongue coating types were investigated. In TCM theories, the tongue coating reflects the status of physiological and clinicopathological changes of inner parts of body. As a common type, thin-white tongue coating is a symbol of good health. A white-greasy tongue coating like powder indicates turbidity and external pathogenic heat. The abundant bacterial groups found in our study are similar to those found in most other studies. Several studies completing microbial analysis of the healthy human tongue using 16S rRNA sequencing showed the most abundant phyla to be Fusobacteria, Actinobacteria, Firmicutes, Proteobacteria, and Bacteroidetes. Jiang et al. investigated 27 tongue coating samples by Illumina technology and identified 715 differentially abundant, species-level OTUs on tongue coatings of the enrolled patients compared to healthy controls. Furthermore, 123 and 258 species-level OTUs were identified in patients with Cold/Hot Syndrome. In Jiang et al.'s report, the dominant phyla in Chinese tongue coating microflora samples were Firmicutes, Bacteroidetes, Proteobacteria, Actinobacteria, and Fusobacteria. The dominant phyla in our six tongue coating samples were Firmicutes, Proteobacteria, Bacteroidetes, Actinobacteria, and Chlorobi. The similar results represented the effectiveness of our methodology compared with other sequencing systems. Han et al. sequenced the V2–V4 region of 16S rRNA gene by pyrosequencing to investigate the tongue coating microbiome in patients with colorectal cancer and healthy controls.* Prevotella*,* Haemophilus*, and* Streptococcus* were dominant in Han et al.'s samples, which is the same result in our study. Based on these conclusions, the method combining ligation-based sequencing and Eco57I digestion exhibited equivalent effect compared with Illumina or pyrosequencing technologies.

Using the 16S rRNA gene, the core function of tongue coating microbiome could be predicted by the PICRUSt software. Pathways encoding for carbohydrate metabolism and oxidative phosphorylation metabolism were detected. The oral cavity is a major gateway to the human body. Microorganisms colonizing in the oral cavity have a significant probability of spreading to the stomach, lung, and intestinal tract. The metagenome prediction validated the role of tongue coating.

In our results, except for some common genera in human body, a few environmental bacteria were observed as well. One of the explanations is that the sequencing results are not precise. The other hypothesis is that these genera are still unknown bacteria, which have similar sequences with the environmental bacteria from database. The more bacteria are sequenced, the more affirmatory genera could be defined.

The composition of the microbial communities on the tongue coating varies between individuals. UniFrac principal coordinates analysis showed no apparent clustering of microbial communities between two types of tongue coating. This may be indicative of the fact that, despite the many different habitats on the human tongue, many bacterial species are shared among those habitats.

## Figures and Tables

**Table 1:** Clinical parameters and barcode primer sequences of study subjects.

Subjects ID	Gender	Age	TCM tongue coating type	Barcode sequence (5′ to 3′)
A4	Male	30	Thin-white	CTGCCCCGGGTTCCTCATTCTCT***AAGCCC***CTGCTGTACGGCCAAGGCG
A5	Male	58	Thin-white	CTGCCCCGGGTTCCTCATTCTCT***CACACC***CTGCTGTACGGCCAAGGCG
A6	Female	72	Thin-white	CTGCCCCGGGTTCCTCATTCTCT***TCCCTT***CTGCTGTACGGCCAAGGCG
B2	Male	57	White-greasy	CTGCCCCGGGTTCCTCATTCTCT***CCGATT***CTGCTGTACGGCCAAGGCG
B3	Female	34	White-greasy	CTGCCCCGGGTTCCTCATTCTCT***TCGTTG***CTGCTGTACGGCCAAGGCG
B4	Male	62	White-greasy	CTGCCCCGGGTTCCTCATTCTCT***GGGCAC***CTGCTGTACGGCCAAGGCG

**Table 2:** The designed multigroup PCR primers.

Primer ID	Genus name	Primer sequence (5′ - 3′)	*T*_m_ (°C)	Amplicon length (bp)
ActF	*Actinomyces*	P^a^-E^b^-GCGAAGAACCTTACCAAGGC	56.2	142
ActR	*Actinomyces*	Q^c^-E-TGACGACAACCATGCACCAC		
PreF	*Prevotella*	P-E-GAACCTTACCCGGGCTTGAA	54.2	138
PreR	*Prevotella*	Q-E-TGACGACAACCATGCAGCAC		
StrF	*Streptococcus*	P-E-AACGATAGCTAATACCGCAT	46.1	139
StrR	*Streptococcus*	Q-E-TAATACAACGCAGGTCCATC		
TreF	*Treponema*	P-E-CGCGAGGAACCTTACCTGGG	53.7	137
TreR	*Treponema*	Q-E-ACGACAGCCATGCAGCACCT		
LepF	*Leptotrichia*	P-E-ACGCGAGGAACCTTACCAGA	53.5	139
LepR	*Leptotrichia*	Q-E-CAGCCATGCACCACCTGTCT		
NeiF	*Neisseria*	P-E-CGGGTTGTAAAGGACTTTTG	49.2	135
NeiR	*Neisseria*	Q-E-AGTTAGCCGGTGCTTATTCT		
SelF	*Selenomonas*	P-E-CTTCGGATCGTAAAGCTCTG	51.4	142
SelR	*Selenomonas*	Q-E-TAGTTAGCCGTGGCTTCCTC		
CapF	*Capnocytophaga*	P-E-TACGCGAGGAACCTTACCAA	51.3	138
CapR	*Capnocytophaga*	Q-E-ACAACCATGCAGCACCTTGA		
PorF	*Porphyromonas*	P-E-CAGCCAAGTCGCGTGAAGGA	54.0	172
PorR	*Porphyromonas*	Q-E-CTGGCACGGAGTTAGCCGAT		
FusF	*Fusobacterium*	P-E-ACGCGTAAAGAACTTGCCTC	51.4	75
FusR	*Fusobacterium*	Q-E-ACGCGTAAAGAACTTGCCTC		
MycF	*Mycoplasma*	P-E-GATGGAGCGACACAGCGTGC	55.2	187
MycR	*Mycoplasma*	Q-E-GCGGCTGCTGGCACATAGTT		
DiaF	*Dialister*	P-E-CGGAATTATTGGGCGTAAAG	53.1	161
DiaR	*Dialister*	Q-E-CTTTCCTCTCCGATACTCCA		
EubF	*Eubacterium*	P-E-GATACCCTGGTAGTCCACGC	51.6	146
EubR	*Eubacterium*	Q-E-CTCCCCAGGTGGAATACTTA		
FirF	*Firmicutes*	P-E-GATACCCTGGTAGTCCACGC	51.6	146
FirR	*Firmicutes*	Q-E-CTCCCCAGGTGGAATACTTA		
PepsF	*Peptostreptococcus*	P-E-TAATTCGAAGCAACGCGAAG	52.7	154
PepsR	*Peptostreptococcus*	Q-E-CGACAACCATGCACCACCTG		
KinF	*Kingella*	P-E-GGAATTACTGGGCGTAAAGC	52.4	168
KinR	*Kingella*	Q-E-AATTCTACCCCCCTCTGACA		
PepnF	*Peptoniphilus*	P-E-ATCACTGGGCGTAAAGGGTT	51.1	186
PepnR	*Peptoniphilus*	Q-E-CGCATTTCACCGCTACACTA		
LachF	*Lachnospiraceae*	P-E-CAACGCGAAGAACCTTACCA	54.2	146
LachR	*Lachnospiraceae*	Q-E-GACGACAACCATGCACCACC		
PasF	*Aggregatibacter* ^b^	P-E-CGGGTTGTAAAGTTCTTTCG	48.4	135
PasR	*Aggregatibacter*	Q-E-TTAGCCGGTGCTTCTTCTGT		
PasF	*Haemophilus*	P-E-CGGGTTGTAAAGTTCTTTCG	48.4	135
PasR	*Haemophilus*	Q-E-TTAGCCGGTGCTTCTTCTGT		
LactF	*Lactobacillus*	P-E-CCGCAACGAGCGCAACCCTT	55.3	103
LactR	*Lactobacillus*	CCTCCGGTTTGTCACCGGCA		
TM7F	*TM7*	P-E-GGGCGTAAAGAGTTGCGTAG	49.2	185
TM7R	*TM7*	Q-E-TACGGATTTCACTCCTACAC		
GemF	*Gemella*	AAAGCTCTGTTGTTAGGGAA	46.8	98
GemR	*Gemella*	P-E-GGTGGCTTTCTGGTTAGGTA		
PseF	*Pseudomonas*	GGCGGCAGGCCTAACACATG	56.5	99
PseR	*Pseudomonas*	P-E-TTACTCACCCGTCCGCCGCT		
VeiF	*Veillonella*	P-E-GTAAAGCTCTGTTAATCGGG	48.1	100
VeiR	*Veillonella*	GTGGCTTTCTATTCCGGTAC		
MogF	*Mogibacterium*	P-E-CACGTGCTACAATGGTCGGT	50.7	101
MogR	*Mogibacterium*	ATCCGAACTGGGATCGGTTT		
CatF	*Catonella*	P-E-CATGCAAGTCGAACGGAGAT	50.8	101
CatR	*Catonella*	GTTACTCACCCGTCCGCCAC		

^a^CCAAGGCGGCCGTACG; ^b^CTGAAG; ^c^CCGACGTCGACTATCCAT.

**Table 3:** Statistical results of SOLiD sequencing tags mapping with RDP references.

Subject ID	Raw reads	Matched reads		Uniquely matched reads	
		Counts	Frequency	Counts	Frequency
A4	12,455,117	2,043,364	16.41%	119,454	0.96%
A5	5,043,037	861,788	17.09%	63,751	1.26%
A6	27,658,749	4,965,785	17.95%	418,203	1.51%
B2	10,171,189	1,406,962	13.83%	160,263	1.58%
B3	8,938,854	1,968,093	22.02%	96,206	1.08%
B4	10,848,548	1,915,504	17.66%	119,292	1.1%

**Table 4:** OTUs assignment of unique tags based on RDP classifier.

OTU	A4		A5		A6		B2		B3		B4	
	Number	PCT (%)	Number	PCT (%)	Number	PCT (%)	Number	PCT (%)	Number	PCT (%)	Number	PCT (%)
Genus	548	93.20	406	94.90	495	95.93	464	94.90	388	94.90	637	95.22
Family	15	2.55	11	2.57	10	1.94	12	2.45	10	2.45	17	2.54
Order	9	1.53	3	0.70	4	0.78	4	0.73	3	0.73	6	0.90
Class	9	1.53	3	0.70	6	1.16	7	1.22	5	1.22	8	1.20
Phylum	1	1.70	0	0	0	0	1	0.24	1	0.24	0	0
Domain	6	1.02	5	1.17	1	0.19	3	0.49	2	0.49	1	0.15
Total OTUs	588		428		516		491		409		669	

**Table 5:** Relative abundance comparison of significantly different genera between thin-white and white-greasy tongue coating types.

Phylum	Genus	A4	A5	A6	B2	B3	B4	*P*
Bacteroidetes	*Proteiniphilum*	85	71	60	0	0	12	**0.003**
Bacteroidetes	*Salinibacter*	228	274	328	68	25	13	**0.004**
Proteobacteria	*Thiobacter*	166	199	226	0	0	10	**0.006**
Firmicutes	*Cryptanaerobacter*	219	320	257	42	16	0	**0.007**
Firmicutes	*Peptococcus*	439	295	338	26	16	0	**0.013**
Firmicutes	*Veillonella*	2047	2501	1434	383	861	111	**0.019**
Bacteroidetes	*Bacteroides*	219	313	188	111	16	26	**0.019**
Firmicutes	*Anaerostipes*	0	0	0	100	168	158	**0.021**
Firmicutes	*Sporobacterium*	9	15	11	0	0	0	**0.022**
Firmicutes	*Fastidiosipila*	11	15	6	31	23	21	**0.026**
Bacteroidetes	*Prevotella*	21	13	15	0	0	0	**0.027**
Firmicutes	*Parasporobacterium*	20	15	11	0	0	0	**0.028**
Proteobacteria	*Smithella*	23	19	22	0	11	0	**0.028**
Firmicutes	*Anaerobacter*	0	0	0	31	39	20	**0.033**
Proteobacteria	*Thioalkalispira*	47	31	24	0	0	0	**0.038**
Firmicutes	*Trichococcus*	127	171	81	4652	5795	9135	**0.041**
Firmicutes	*Faecalibacterium*	1504	2974	1936	95	47	232	**0.042**
Firmicutes	*Lachnobacterium*	106	79	47	0	0	0	**0.046**
Bacteroidetes	*Subsaxibacter*	0	17	0	26	54	65	**0.047**
